# Correction: Anaerobic oxidation of aldehydes to carboxylic acids under hydrothermal conditions

**DOI:** 10.1039/d3ra90014b

**Published:** 2023-02-22

**Authors:** Yiju Liao, Alexandria Aspin, Ziming Yang

**Affiliations:** a Department of Chemistry, Oakland University Rochester MI 48309 USA zimingyang@oakland.edu

## Abstract

Correction for ‘Anaerobic oxidation of aldehydes to carboxylic acids under hydrothermal conditions’ by Yiju Liao *et al.*, *RSC Adv.*, 2022, **12**, 1738–1741, https://doi.org/10.1039/D1RA08444E.

The authors regret that the incorrect structure was shown for Compound **5** in Table 2. The corrected version of Table 2 is shown below.

**Table:** Investigation of substrate scope under anaerobic hydrothermal conditions of 200 °C, 15 bar after 2 h

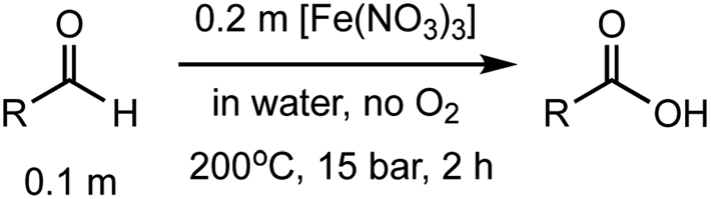				
Comp#	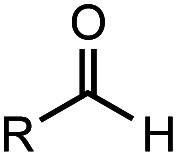	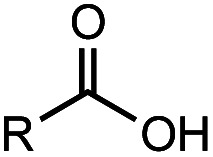	Conversion	Acid yield^a^
**1**	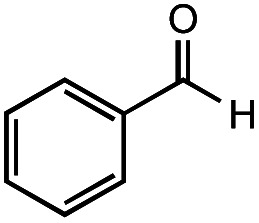	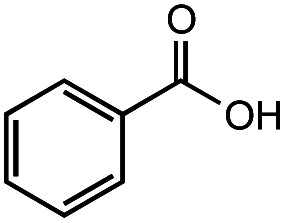	>99%	98%
**2**	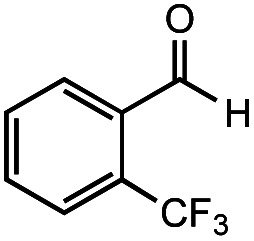	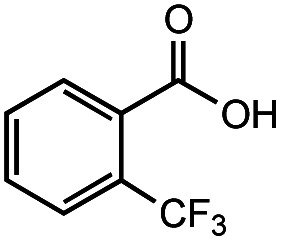	83%	82%
**3**	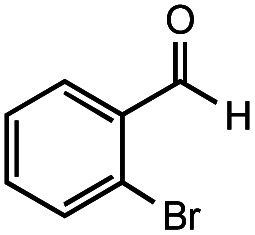	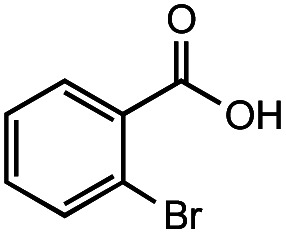	56%	47%
**4**	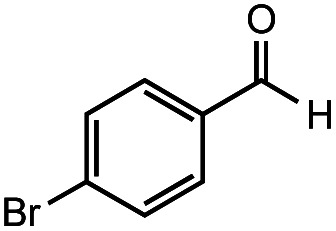	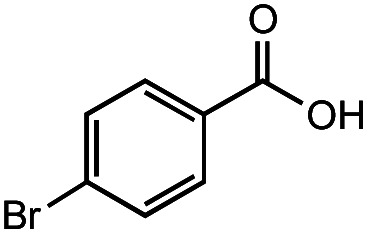	68%	66%
**5**	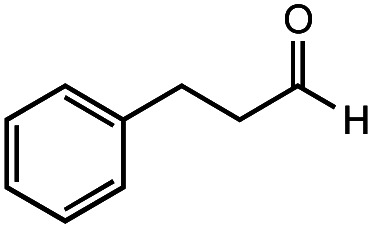	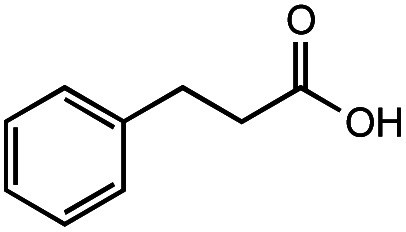	66%	45%
**6**	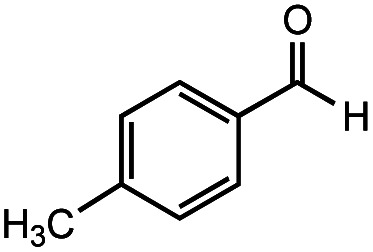	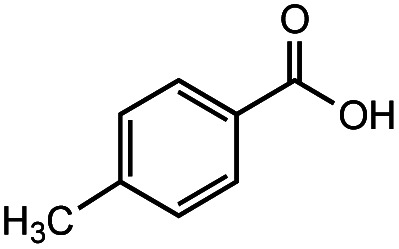	55%	54%
**7**	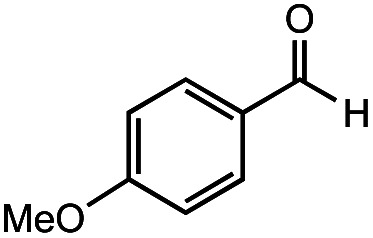	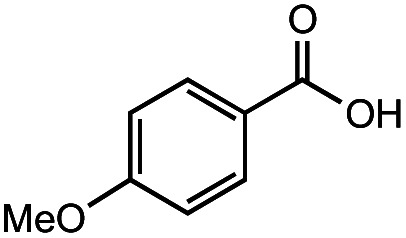	63%	46%
**8**	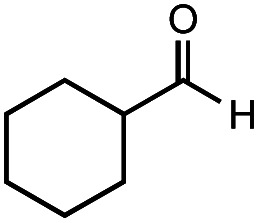	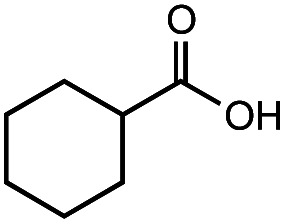	66%	58%
**9**	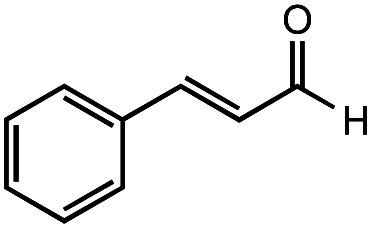	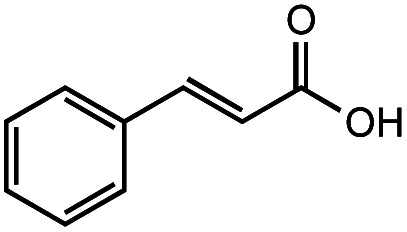	48%	30%
**10**	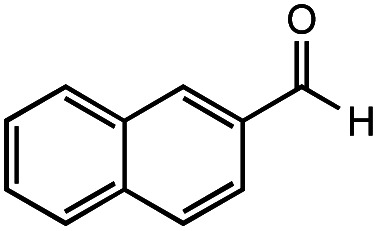	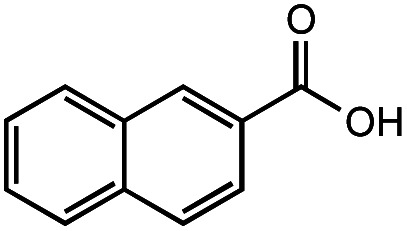	42%	41%

a Yield determined by gas chromatography.

The Royal Society of Chemistry apologises for these errors and any consequent inconvenience to authors and readers.

## Supplementary Material

